# Fuzheng Kang-Ai decoction enhances the effect of Gefitinib-induced cell apoptosis in lung cancer through mitochondrial pathway

**DOI:** 10.1186/s12935-020-01270-3

**Published:** 2020-05-24

**Authors:** Sumei Wang, Zhiwei Peng, Wenjuan Li, Shunqin Long, Shujing Xiao, Wanyin Wu

**Affiliations:** 1 Department of Oncology, Clinical and Basic Research Team of TCM Prevention and Treatment of NSCLC, Guangdong Provincial Hospital of Chinese Medicine, The Second Clinical Medical College of Guangzhou University of Chinese Medicine, No. 111, Dade Road, Guangzhou, 510120 Guangdong People’s Republic of China; 2 The Postdoctoral Research Station, Guangdong Provincial Hospital of Chinese Medicine, Guangzhou University of Chinese Medicine, Guangzhou, 510120 Guangdong People’s Republic of China; 3 The Basic Medicine College, Guangzhou University of Chinese Medicine, Guangzhou, 510002 Guangdong People’s Republic of China; 4 School of Nursing, Guangzhou Kangda Vocational Technical College, Guangzhou, 511363 Guangdong People’s Republic of China; 5 Guangdong Provincial Key Laboratory of Clinical Research on Traditional Chinese Medicine Syndrome, Guangzhou, 510120 Guangdong People’s Republic of China

**Keywords:** FZKA decoction, Gefitinib, Lung cancer, Apoptosis, Mitochondrial pathway

## Abstract

**Background:**

Our previous clinical study has shown that Chinese herbal medicine (CHM) Fuzheng Kang-Ai (FZKA) decoction is effective in treating advanced lung cancer patients through prolonging the drug resistance to Gefitinib (GFTN). Our basic study found that FZKA decoction could enhance the inhibition effect of GFTN in lung cancer by inactivating PI3K/Akt pathway. Moreover, our recent work showed that FZKA induced lung cancer cell apoptosis via STAT3/Bcl-2/Caspase-3 pathway. Thus in this study, we aim to elucidate how FZKA enhances the effect of GFTN in lung cancer from the perspective of cell apoptosis.

**Methods:**

Cell proliferation and colony formation assay were performed to detect the cell growth inhibition. Flow cytometry and TUNEL assay were carried out to test the cell apoptosis. Mitochondrial membrane potential (MMP) assay was done to measure the alteration of MMP. Caspase-3/-9 activity assay was used to test the activity of caspase-3/-9. Western blot and qRT-PCR were done to detect the expression of STAT3 and Bcl-2 family as well as Caspase-3/-9 and Cyt-*C* at protein and mRNA levels, respectively. Transient transfection was performed to silence STAT3 using siSTAT3. Animal model was done to validate the molecular mechanisms in vivo and immunohistochemistry was done to detect the expression of Bax and Caspase-3.

**Results:**

Firstly, our results showed that FZKA enhanced the inhibition effect of GFTN in lung cancer both in vitro and in vivo. Secondly, cell apoptosis was enhanced when treating lung cancer cells with both FZKA and GFTN, a process involving the mitochondria and the Bcl-2 family. And Bcl-2 family was involved in this process. Interestingly, STAT3 plays a critical role on mediating the above process. Last but not the least, the enhanced effect of cell apoptosis induction of GFTN by FZKA was validated in animal model.

**Conclusion:**

Our findings conclude that Fuzheng Kang-Ai decoction enhances the effect of GFTN-induced cell apoptosis in lung cancer through the mitochondrial pathway, providing a novel molecular mechanism by which FZKA sensitizes to GFTN by delaying drug resistance in treating lung cancer patients.

## Background

Nowadays, we still face serious health damage from lung cancer, which is a leading cause of cancer-related death in the world [1]. In China, the incidence of lung cancer has grown rapidly, resulting in a large social and economic burden [2]. Gefitinib is an effective selective inhibitor of epidermal growth factor receptor tyrosine kinase (EGFR-TKI), which has been approved for treating advanced non-small cell lung cancer (NSCLC) patients in the first line, showing better clinical efficacy than chemotherapy [3, 4]. However, the main obstacle restricting the clinical use of Gefitinib is the acquired drug resistance. And patients with NSCLC will acquire drug resistance to Gefitinib around 1 year after treating with Gefitinib [5]. Thus, urgent strategies are necessary to resolve the acquired drug resistance to Gefitinib.

In recent studies, we found that the Chinese herbal medicine (CHM) Fuzheng Kang-Ai (FZKA) decoction can to some extent increase the efficacy of Gefitinib and reduce its drug toxicity, which is a promising strategy for treating patients with advanced NSCLC. FZKA decoction, pioneered by Wanyin Wu, is a prescription composed of 12 traditional Chinese medicines and has been widely used in the clinical treatment of NSCLC in recent decades in Guangdong Provincial Hospital of Traditional Chinese Medicine [6]. We previously reported that FZKA could prolong the progression-free survival (PFS) and reduce the toxic effect of NSCLC patients when treated with Gefitinib and FZKA both [7, 8]. And FZKA could also enhance the disease control rate (DCR) as well as median survival time (MST) in patients with NSCLC [9, 10]. Mechanistically, we previously found that FZKA decoction could inhibit the growth of lung cancer cells through AMPKα/IGFBP1/FOXO3a, and PI3-K/Akt/NF-κB pathways, demonstrating its definite effect in treating lung cancer [11, 12]. The above study showed that FZKA could inhibit the cell growth of lung cancer. Recently, we’ve found that FZKA decoction promotes lung cancer cell apoptosis [13]. Therefore, we hypothesized that FZKA decoction might enhance the effect of Gefitinib in lung cancer by regulating cell apoptosis.

In this study, we showed that there is a synergistic effect of FZKA decoction and Gefitinib in lung cancer. Both our in vitro and in vivo studies have provided a potential novel mechanism by which the FZKA decoction enhances the cell apoptosis of Gefitinib in lung cancer. To test our hypothesis, we performed flow cytometry, Caspase activity assay and mitochondrial membrane potential assay as well as TUNEL assay and etc. to elucidate the molecular mechanism by which FZKA enhances the effect of Gefitinib in lung cancer. We conclude that FZKA decoction enhances the effect of Gefitinib-induced cell apoptosis in lung cancer through mitochondrial pathway. Our findings provided a valid evidence for the clinical application of traditional medicine FZKA decoction in curing lung cancer patients, especially for the NSCLC patients treated with Gefitinib.

## Materials and methods

### Fuzheng Kang-Ai decoction (FZKA decoction)

FZKA decoction, a Chinese herbal medicine (CHM) prescribed at the clinic, obtained from Guangdong Kangmei Pharmaceutical Company Ltd (Guangdong, China), has been used to treat NSCLC in Guangdong Provincial Hospital of Traditional Chinese Medicine in recent decades. We listed the components of FZKA decoction in Table 1, and it has been reported previously [10]. The batch to batch consistency studies (the chromatograms in FZKA decoction) using high performance liquid chromatography (HPLC) analysis, chemical profiling of main constituents in FZKA decoction using ultra-high pressure liquid chromatography coupled with LTQ Orbitrap mass spectrometry have been reported previously and are shown in Additional file 1: Figure S1 [11]. All of the components of this prescription were soaked for 30 min before decoction. The concentration liquid was finally spray dried into particles by Guangdong one Pharmaceutical Co., Ltd. For in vitro experiments, the granules were dissolved in RPMI-1640 medium to a final concentration of 20 mg/mL and centrifuged at 14,000 rpm for 10 min; the supernatant was then filtered with 0.22 μm filter before use and the pH value of the cultured cells with media was adjusted to 7.2–7.4 after FZKA addition. For in vivo experiments, animals are treated with FZKA decoction by intragastric administration (Table 1).

**Table 1:** Components of “Fuzheng Kangai” (FZKA) decoction

Chinese name	Common name	Weight (g)
Taizishen	Radix Pseudostellariae	30
Baizhu	Rhizoma Atractylodis Macrocephalae	15
Huangqi	Milkvetch Root	30
Baihuasheshecao	Hedyotis Diffusa	30
Longkui	Solanum Nigrum	30
Shijianchuan	Chinese Sage Herb	30
Shancigu	Indian Iphigenia Bulb	30
Yiyiren	Coix Seed	30
Bayuezha	Akebia Trifoliata Koidz	30
Shepaole	Snake Bubble Ilicifolius	30
Ezhu	Curcuma Zedoaria	15
Gancao	Licorice	10

### Chemicals and cell culture

Monoclonal antibodies specific of Caspase-3 (cleaved Caspase-3), Caspase-9 (cleaved Caspase-9), Bcl-2, myeloid cell leukemia-1 (Mcl-1), Bax, Bim, Cytochrome *C* (Cyt-*C*), and STAT3 were purchased from Cell Signaling Technology Inc. (Beverly, MA, USA). Caspase-3 and Caspase-9 activity assay kits were ordered from Abcam (Cambridge, MA, USA). Lipofectamine 3000 reagent was purchased from Life Technologies (Carlsbad, CA, USA). SiSTAT3 (#1,2,3) and all qRT-PCR primers including Bcl-2, Mcl-1, Bax, Bim, STAT3 and GAPDH were purchased from Ribobio (Guangzhou, Guangdong, China). All primer sequences and siSTAT3 target sequences were shown in Additional file 1: Table S1. NSCLC cells A549 were obtained from the Cell Line Bank at the Laboratory Animal Center of Sun Yat-sen University (Guangzhou, China) and PC9 were obtained from the Chinese Academy of Sciences Cell Bank of Type Culture Collection (Shanghai, China). All cells were grown at 37 °C, in a humidified 5% CO_2_ and 95% air and cultured in RPMI-1640 medium (Life Technologies, Carlsbad, CA, USA) containing 10% FBS (Gibco, USA) and 0.5% penicillin–streptomycin sulfate (Invitrogen, Carlsbad, CA, USA). Cells were counted using the automated cell counter star (Invitrogen, Carlsbad, CA, USA).

### High performance liquid chromatography (HPLC)

The initial batch to batch consistency study was performed using HPLC, as previously reported [11]. Briefly, the samples solutions were put into the HPLC system (250 × 4.6 mm, 5 μm, ACE, Scotland). The mobile phase consisted of deionized water with 0.1% formic acid (A) and acetonitrile with 0.1% formic acid (B). The gradient elution program was as follows: 5% B at 0–5 min, 5–20% B at 5–10 min, 20–40% B at 10–15 min, 40–95% B at 15–40 min, and 95–100% B at 40–45 min. The flow rate was 1.0 mL/min, and the detection wavelength was set at 280 nm. The injection volume was 10 μL and the column temperature was maintained at 30 °C.
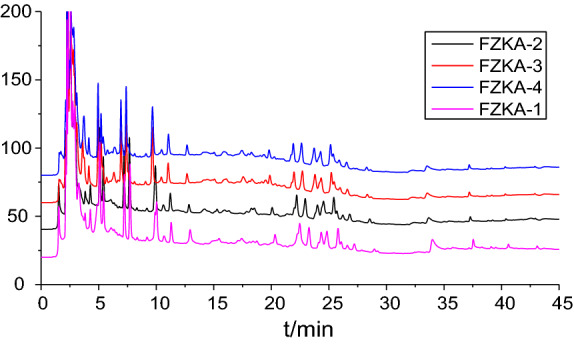


### Cell proliferation assay

Cell proliferation was done and analyzed using the RTCA xCELLigence system. Briefly, 100 μL cells were plated into E-plate 16 (with 50 μL medium per well). The E-plate has been already set in the RTCA station as step 1 (sweeps 1, Interval 1 min, Total time 1 min). Then the E-plate 16 was put into the RTCA station after keeping in the laminar flow cabinet for 30 min. And the RTCA was set as step 2 (sweeps 180, Interval 30 min, Total time 89 h). FZKA and Gefitinib were added to the E-plate 16 after cell incubation for 24 h. The cell analyzer is placed in the CO^2^ incubator, and the computer is equipped with xCELLigence RTCA S16 software. This computer was used to control the operation of the whole system and to conduct data analysis.

### Colony formation assay

Colony formation assay was performed to study the inhibition effect of FZKA and Gefitinib in NSCLC cells. Briefly, 200 cells were plated into 6-well plate. FZKA and Gefitinib were added after 24 h for cell incubation. Cell medium was changed after 24 h. Around 8–10 days, cell colonies were washed with PBS, fixed with methanol, stained with 0.1% crystal violet, and scored by counting the number of colonies with an inverted microscope. The inhibition ratios (%) of colony formation were calculated as the ratio of the indicated treatment group to the control group as follows: % inhibition ratio = 100% × Nt/Nc, where Nt is the number of the treatment group colonies and Nc is the number of control group colonies.

### Animal model

For the xenograft tumor model, the right flank of 4- to 6-week-old female BALB/c athymic nu/nu mice (from Beijing vital river experimental animal Co. Ltd) were subcutaneously injected with 100 μL 1.0 × 10^6^ NSCLC (PC9-luc and A549-luc, respectively) cells carrying luciferase report gene. The animal experiments were approved by Institutional Animal Care and Use Committee of Guangdong Provincial Hospital of Chinese Medicine (Ethics Approval Number 2018020). When the tumor mass became palpable (at day 4 after injection), mice were randomly divided into four groups: control, FZKA (FZKA was given by gavage), Gefitinib (Gefitinib was given by intraperitoneal injection) and combination with FZKA and Gefitinib. Tumors were measured every 2 days with digital calipers. The tumor volume (in mm^3^) was calculated using the formula: Volume = (L × W^2^)/2. Mice were sacrificed around day 15 after injection (PC9-luc mice were killed on day 11 after injection), when some of the tumors reached the size limit set by the institutional animal care and use committee. Tumors were weighed after careful resection. In addition, bioluminescence imaging (BLI) was performed before and after treating mice with drugs. Briefly, mice were anesthetized by inhalation of 2% isoflurane. Mice were injected intraperitoneally with 150 mg/kg d-luciferin (Caliper, PerkinElmer, Waltham, MA, USA) in approximately 100 μL. The intensity of BLI signal was determined using the IVIS-200 Imaging System (Xenogen, Alameda, CA, USA).

### Flow cytometry analysis

Cell apoptosis was performed using Annexin V-FITC/PI apoptosis detection kit according to the manufacturer’s protocol (Sigma-Aldrich Co. St. Louis, MO), as previously performed [13]. In brief, NSCLC cells (A549 and PC9) were grown in 6-well plates. After 24 h of culture, cells were treated with FZKA and Gefitinib in different groups and then incubated at 37 °C for 24 h. Afterwards, cells were collected, centrifuged for 5 min at 1500 rpm, and resuspended in 1× binding buffer. Finally, 5 μL Annexin V-FITC and 5 μL PI were added into the cells at room temperature for 15 min. The cells were then analyzed using flow cytometer (Beckman FC 500, Beckman Coulter, Inc, CA, USA).

### Caspase-3/9 activity assay

Caspase-3/9 activities were measured using Caspase-3/9 activity assay kit according to manufacturer’s protocol (Cell Signaling Technology Inc. Beverly, MA, USA), as previously performed [13]. Briefly, NSCLC cells (A549 and PC9) treated with FZKA and Gefitinib were harvested and lysed in lysis buffer (4 °C for 30 min). Equal amounts of formulations (100 μL) were loaded onto a 96-well microplate and incubated at 37 °C for 120 min with reaction buffer and the liberated DEVD-p-NA substrate. Then the Caspase3/9 activity was measured using the microplate reader at a wavelength of 405 nm. The Caspase3/9 activities were expressed as percentage of enzyme activity compared to that in the control group (untreated cells).

### Western blot analysis

Western blot was performed as previously reported [13]. Briefly, the cells were harvested, washed and lysed with 1× RIPA buffer. Thermo BCA protein assay kit was used to test the protein concentration. Equal amounts of protein from cell lysates or animal tissue were solubilized in 5× SDS sample buffer and separated on 8–10% SDS polyacrylamide gels, and transferred onto polyvinylidene fluoride (PVDF) membranes. Membranes were blocked with 5% non-fat milk in TBST and incubated with primary antibodies against Caspase-3, Caspase9, Cyt-*C*, Bcl-2, Mcl-1, Bax, Bim and STAT3 proteins at 4 °C overnight. Then the membranes were washed and incubated with a secondary antibody against rabbit IgG for 1 h, followed by washing and transferring into ECL solution (Millipore, Darmstadt, Germany), and scanned using the Bio-Rad ChemiDoc XRS+ Chemiluminescence imaging system (Bio-Rad, Hercules, CA, USA). All the results were analyzed by Image J software.

### Mitochondrial membrane potential (MMP) assay

Mitochondrial membrane potential assay was performed using JC-1 kit according to the manufacture’s protocol (Beyotime Biotechnology, Shanghai, China). Briefly, cells were harvested and the JC-1 staining solution was added. After a 20 min incubation at 37 °C, it was washed twice with JC-1 staining buffer and observed under a fluorescence microscope. If the mitochondrial membrane potential was high, JC-1 accumulated in the mitochondrial matrix, forming a polymer and producing red fluorescence. While when the mitochondrial membrane potential was low, JC-1 could not aggregate in the mitochondrial matrix, but existed as JC-1 monomer, resulting in green fluorescence. So the change of mitochondrial membrane potential can be detected by the change of fluorescence color. Photos were taken by fluorescence microscope (Nikon). The excitation wavelength of JC-1 monomer is 514 nm, and of J-aggregates is 585 nm. The emission wavelength of JC-1 monomer is 529 nm, and of J-aggregates is 590 nm. The fluorescence intensity was detected using multimode reader (Tecan infinite M1000pro, Dicken, Austria).

### Quantitative real-time polymerase chain reaction (qRT-PCR)

qRT-PCR was used to detect mRNA levels. RNA was extracted from cells using Trizol (Ambion). Transcriptor first strand cDNA synthesis kit (Roche, Basel, Switzerland) was used to convert RNAs to cDNAs. And FS essential DNA green master (Roche, Basel, Switzerland) was used to perform qRT-PCR in a LightCycler 480 multiwell plate 96 by LightCycler^®^ 96 SW 1.1 (Roche). Cycling variables were as follows: 95 °C for 10 min followed by 40 cycles at 95 °C (10 s) and annealing/extension at 60 °C (20 s)/72 °C (20 s). Data were analyzed with 2^−ΔΔCt^ for relative changes in gene expression. GAPDH was used as an internal control.

### Transient transfection assays

The cells were seeded in 6-well plates and reached to 30–50% confluence. The control (C, si-GAP, NC) and si-STAT3 (#1,2,3) overexpression vectors were obtained from RiboBio (Guangzhou, China). In si-STAT3 group, 50 nM STAT3 siRNA were transfected into the cells using CP Regent for 24 h based on the instruction from the provider.

### Immunohistochemistry

Bax and Caspase-3 expression at the protein level was detected immunohistochemically on tumor tissues from mice. Briefly, sections were treated with 10 mM sodium citrate buffer (pH 6.0) for heat-induced retrieval of the antigen and immersed in 3% hydrogen peroxide solution to inhibit endogenous peroxidase activity, followed by incubation of the sections in 5% bovine serum albumin to block nonspecific binding. The sections were incubated with primary antibodies against Bax and Caspase-3 (1:100) at 4 °C overnight and then incubated with biotinylated secondary antibody followed by the Liquid DAB Substrate Chromogen System according to the manufacturer’s instructions. Protein expression level was evaluated by counting at least 500 tumor cells in at least five representative high-power fields. The Bax and Caspase-3 staining in the tumor cells were scored separately and scores combined for each case as described previously [14].

### TUNEL assay

TUNEL assay was processed using the in situ cell death detection kit (Roche, Mannheim, Germany). Treated cells were permeabilizated with proteinase K for 10 min after washing with PBS for three times. Cells were then incubated in TdT buffer at 37 °C for 1 h after another three times of washing. Afterwards, incubated the cells with primary antibody CRIP1 at 4 °C overnight, followed by the incubation of PE-conjugated secondary antibodies and DAPI counterstaining. The apoptosis index was calculated by counting the number of TUNEL/CRIP1-positive cells using a fluorescence microscope (Nikon). For the quantification of each slide, at least five representative high-power fields were calculated. And the final positive cells were the average from three different mice tumor tissues.

### Statistical analysis

Statistical analysis was performed using the SPSS statistical software. Statistical evaluation for data analysis used Student’s t-test when there were only two groups (two sided) and differences between groups were assessed by one-way ANOVA. All data are reported as Mean ± SD. Differences between groups were considered significant statistically when p ≤ 0.05.

## Results

### FZKA decoction enhanced the effect of Gefitinib on lung cancer both in vitro and in vivo

Before this study, we performed high performance liquid chromatography (HPLC) to identify the main components in the different drinks of FZKA decoction. Our results showed that HPLC chromatograms in four drinks of FZKA decoction have similar patterns, revealing that the FZKA decoction has a good stability (Additional file 1: Figure S1 [13]).

**Fig. 1:**
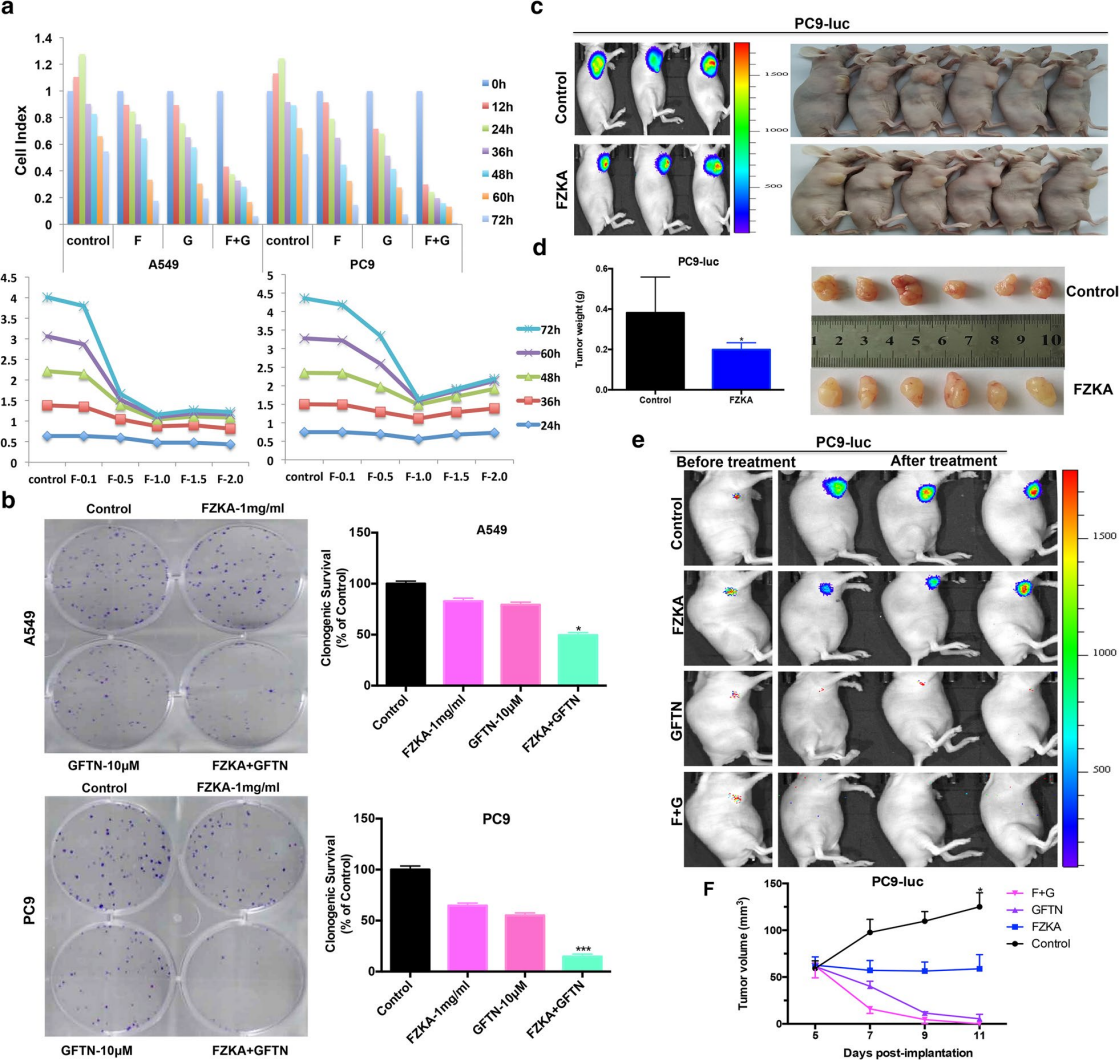
FZKA sensitized the inhibition effect of Gefitinib on lung cancer both in vitro and in vivo. **a** Lung cancer cell growth was the most inhibited in the combination group when combined FZKA with Gefitinib. Lung cancer cells (A549 and PC9) were treated with FZKA, Gefitinib, or FZKA combined with Gefitinib, respectively, and cell proliferation assay was performed to detect the cell viability real-time. **b** Cell colony formation ability was the most inhibited in the combination group when combined FZKA with Gefitinib. Colony formation assay was performed in the lung cancer cells (A549 and PC9) when treated with FZKA, Gefitinib, or FZKA combined with Gefitinib, respectively. Data represent mean ± SD of three independent experiments. *p < 0.05, ***p < 0.0001; one-way ANOVA. **c**, **d** Mice tumor growth generated from lung cancer cell was inhibited by FZKA treatment. PC9 cell carrying luciferase was injected to the nude mice subcutaneously and was then treated with FZKA by intragastric administration. *p < 0.05; Student’s t-test. **e**, **f** Bioluminescence imaging (BLI) photos showing the combination of FZKA and Gefitinib group had a additive inhibition effect on lung cancer tumor growth compared to FZKA along or Gefitinib alone group. And tumor volume was measure every other day until the mice tumor in the combination group was totally inhibited. *p < 0.05; one-way ANOVA

We first performed the cell proliferation assay to identify the synergistic effect of FZKA and Gefitinib on lung cancer cell growth. As shown in Fig. 1a, in both A549 and PC9 cells, cell proliferation was greatly inhibited in the combination group (FZKA combined with Gefitinib) compared to FZKA or Gefitinib group, separately, in a dose- and time-dependent manner. Then the colony ability was detected by colony formation assay and the results reconfirmed that the colony ability was also significantly in the combination group (FZKA combined with Gefitinib) compared to FZKA or Gefitinib group, separately (Fig. 1b). To better observe the synergistic effect of the combination of FZKA with Gefitinib, we then did the animal model and found that FZKA could inhibit the tumor growth with statistical significance (Figs. 1c, d and 6a). Interestingly, since PC9 is a Gefitinib-sensitive cell line, the mice treated with Gefitinib alone had an obvious tumor growth inhibition. However, we still can see a trend that the combination group had a synergistic effect on tumor growth in vivo by bioluminescence imaging (Fig. 1e). Therefore, the above results suggested that there is a synergistic effect of FZKA together with Gefitinib on NSCLC cell growth inhibition both in vitro and in vivo.

### FZKA enhances the effect of Gefitinib-induced lung cancer cell apoptosis

**Fig. 2:**
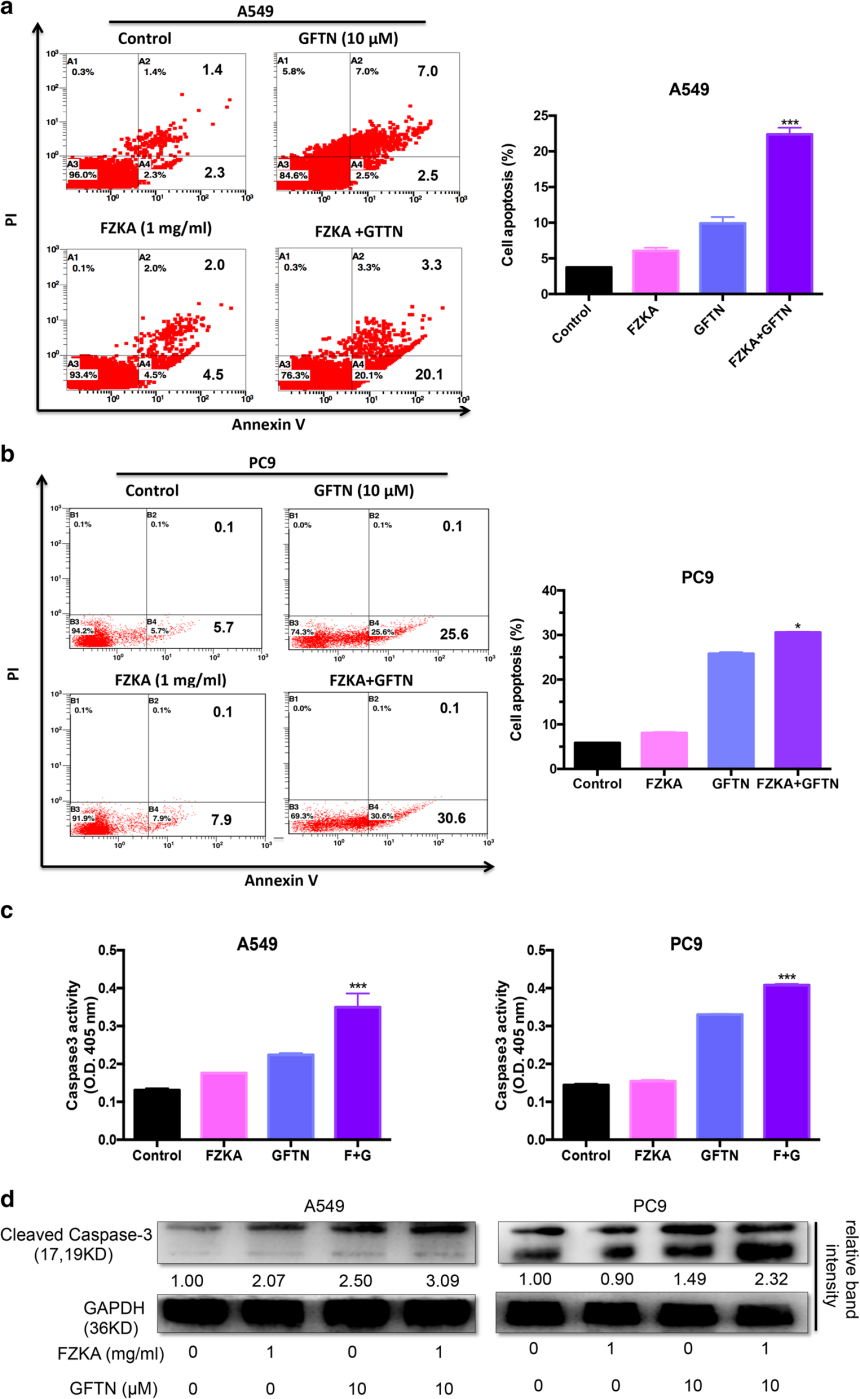
FZKA enhances the effect of Gefitinib-induced lung cancer cell apoptosis. **a**, **b** Cell apoptosis was the most induced in the combination group when treated with FZKA together with Gefitinib. Flow cytometry was performed to detect the cell apoptosis in lung cancer cells (A549 and PC9) when treated with 1 mg/mL FZKA, 10 μM Gefitinib, or FZKA combined with Gefitinib for 24 h, respectively. *p < 0.05, ***p < 0.0001; one-way ANOVA. Data represent mean ± SD of three independent experiments. **c** Caspase-3 activity was the most increased in the combined group treated with FZKA together with Gefitinib. Caspase-3 activity assay was carried out in the lung cancer cells (A549 and PC9) treated with FKZA, Gefitinib, or FZKA combined with Gefitinib for 24 h, respectively. ***p < 0.0001; one-way ANOVA. Data represent mean ± SD of three independent experiments. **d** Cleaved Caspase-3 was the most increased in the combined group treated with FZKA and Gefitinib. Western blot was performed to detect the expression of cleaved Caspase-3 in lung cancer cells (A549 and PC9) treated with FKZA, Gefitinib, or FZKA combined with Gefitinib for 24 h, respectively. GAPDH was used as a loading control. Densitometric analysis was performed using ImageJ

To further study the synergistic effect of FZKA and Gefitinib, flow cytometry of cell apoptosis was performed when treated NSCLC cells (A549 and PC9) with FZKA, Gefitinib, or FZKA together with Gefitinib. Our results showed an obvious synergistic effect of FZKA combination with Gefitinib on lung cancer cell apoptosis induction with statistical significance, especially in A549 cells (Fig. 2a, b). Afterwards, we detected the Caspase-3 activity to identify the cell apoptosis induction effect by FZKA and Gefitinib using Caspase activity assay. As shown in Fig. 2c, the Caspase-3 activity was increased in the combination group compared to the FZKA alone or Gefitinib alone group, respectively. Furthermore, we also observed the same result on the protein expression of cleaved Caspase-3, as shown in Fig. 2d. Those findings suggested that FZKA could enhance the effect of Gefitinib-induced lung cancer cell apoptosis. How FZKA decoction enhances the effect of Gefitinib in inducing lung cancer cell apoptosis?

### Mitochondrial pathway is involved in FZKA enhanced Gefitinib-induced cell apoptosis

**Fig. 3:**
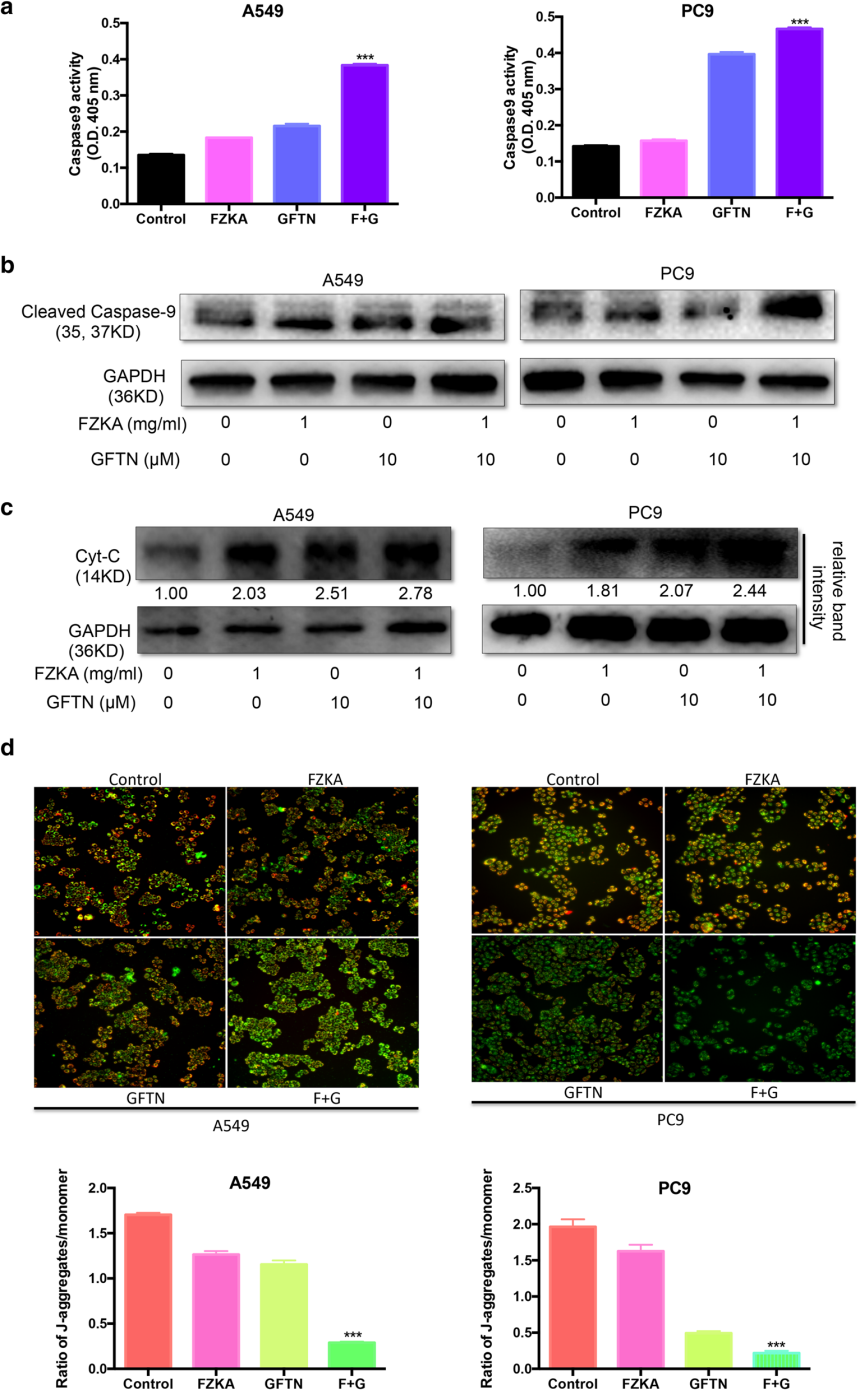
Mitochondrial pathway plays an important role in the enhancing effect of FZKA on cell apoptosis by Gefitinib. **a** Caspase-9 activity was dramatically increased in the combined group treated with FZKA and Gefitinib. Caspase-9 activity assay was done in the lung cancer cells (A549 and PC9) treated with FKZA, Gefitinib, or FZKA combined with Gefitinib for 24 h, respectively. ***p < 0.0001; one-way ANOVA. Data represent mean ± SD of three independent experiments. **b**, **c** Both cleaved-Caspase-9 and Cyt-*C*, two critical proteins in the mitochondrial apoptotic pathway, were the most expressed in the combined group treated with FZKA and Gefitinib. Western blot was performed to detect the cleaved Caspase-9 and Cyt-*C* expression in lung cancer cells (A549 and PC9) treated with FKZA, Gefitinib, or FZKA combined with Gefitinib for 24 h, respectively. GAPDH was used as a loading control. Densitometric analysis was performed using ImageJ. **d** Mitochondrial membrane potential (MMP) was reduced the most in the combined group treated with FZKA and Gefitinib. MMP assay was carried out in lung cancer cells (A549 and PC9) treated with FKZA, Gefitinib, or FZKA combined with Gefitinib for 24 h, respectively. Representative photos were shown as indicated (top panel). And the ratios of J-aggregates/monomer were shown as column diagrams (bottom panel). The red fluorescence means J-aggregates when the MMP is high, and the green fluorescence means monomer when the MMP is low. ***p < 0.0001; one-way ANOVA. Data represent mean ± SD of three independent experiments

To elucidate in which way FZKA increases the effect of Gefitinib-induced cell apoptosis, we detected the activity of Caspase-9, a key component of intrinsic apoptosis pathway (mitochondrial pathway), using Caspase-9 activity assay. Intriguingly, we found that the Caspase-9 activity was increased in the combination group (FZKA together with Gefitinib) compared to the FZKA alone or Gefitinib alone group, respectively (Fig. 3a). In line with this, the same result on the expression of cleaved Caspase-9 protein was observed by Western blot analysis (Fig. 3b). Moreover, Cytochrome *C* (Cyt-*C*) as the critical protein in the mitochondrial pathway had an increased expression level in the combination group (FZKA together with Gefitinib) compared to FZKA or Gefitinib alone group (Fig. 3c). Based on the above results, we then performed mitochondrial membrane potential (MMP) assay to measure the MMP in all the four groups: Control, FZKA alone, Gefitinib alone and the combination group (FZKA combined with GFTN). As shown in Fig. 3d, compared with the FZKA or Gefitinib group, MMP was greatly decreased in the combination group. All in all, these findings indicated that mitochondrial pathway is involved in FZKA enhanced Gefitinib-induced lung cancer cell apoptosis.

### Bcl-2 family was involved in the FZKA enhanced Gefitinib-induced cell apoptosis

**Fig. 4:**
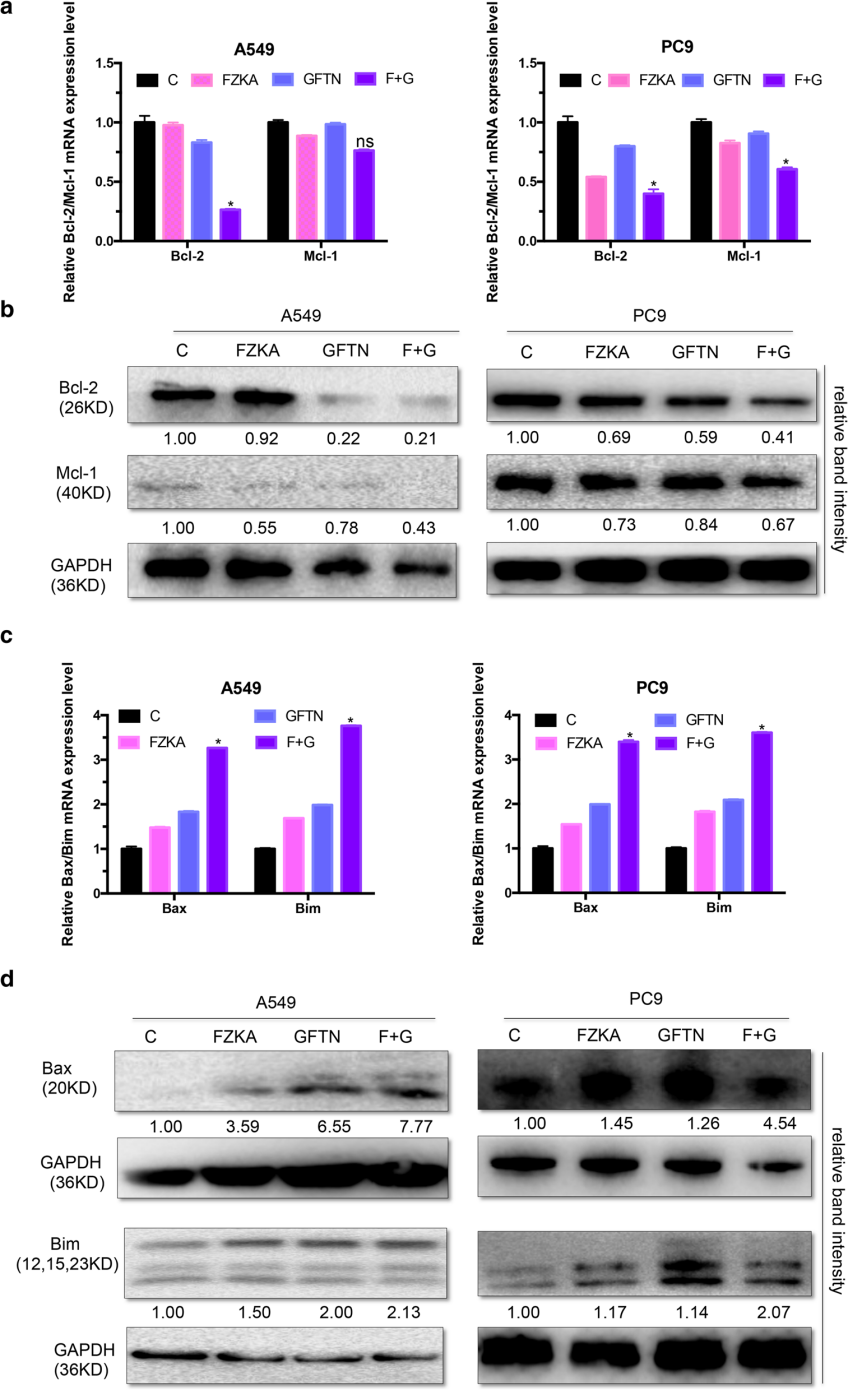
Bcl-2 family was involved in FZKA enhanced effect of cell apoptosis induced by Gefitinib. **a**, **b** The expression levels of Bcl-2 and Mcl-2, two anti-apoptotic proteins, were obviously downregulated in the combined group treated with FZKA and Gefitinib, both at mRNA and protein levels. qRT-PCR and western blot were done to test the expression of Bcl-2 and Mcl-2 at mRNA and protein levels in lung cancer cells (A549 and PC9) treated with FKZA, Gefitinib, or FZKA combined with Gefitinib for 24 h, respectively. *ns* not significant, *p < 0.05; one-way ANOVA. **c**, **d** The expression levels of Bax and Bim, two pro-apoptotic proteins, were obviously upregulated in the combined group treated with FZKA and Gefitinib, both at mRNA and protein levels. qRT-PCR and western blot were done to test the expression of Bax and Bim at mRNA and protein levels in lung cancer cells (A549 and PC9) treated with FKZA, Gefitinib, or FZKA combined with Gefitinib for 24 h, respectively. *p < 0.05; one-way ANOVA. The qRT-PCR data were analyzed with 2^−ΔΔCt^ for relative changed in gene expression. GAPDH was used as an internal control. Densitometric analysis was performed using ImageJ

The apoptosis signaling can be initiated within the cell via the release of pro-apoptotic molecules, such as Bcl-2 family proteins, the key regulator of apoptosis in cancer [15]. To further clarify the molecular mechanisms underlying Gefitinib-induced apoptosis enhanced by FZKA in lung cancer, the expression of Bcl-2 family members including pro-apoptotic proteins (Bcl-2 and Mcl-1 are pro-apoptotic) and anti-apoptotic proteins (Bax and Bim are anti-apoptotic) in A549 and PC9 cells treated with FZKA and Gefitinib were examined. The results demonstrated that the expression of anti-apoptotic proteins (Bcl-2 and Mcl-1) were decreased whereas pro-apoptotic proteins (Bax and Bim) were increased in the combined treatment group, both in a mRNA level by qRT-PCR and protein level by Western blot (Fig. 4a–d). Therefore, the above results indicated that Gefitinib-induced apoptosis in lung cancer sensitized by FZKA might be mediated through Bcl-2 family.

### STAT3 mediates the function of FZKA on enhancing Gefitinib-induced cell apoptosis

**Fig. 5:**
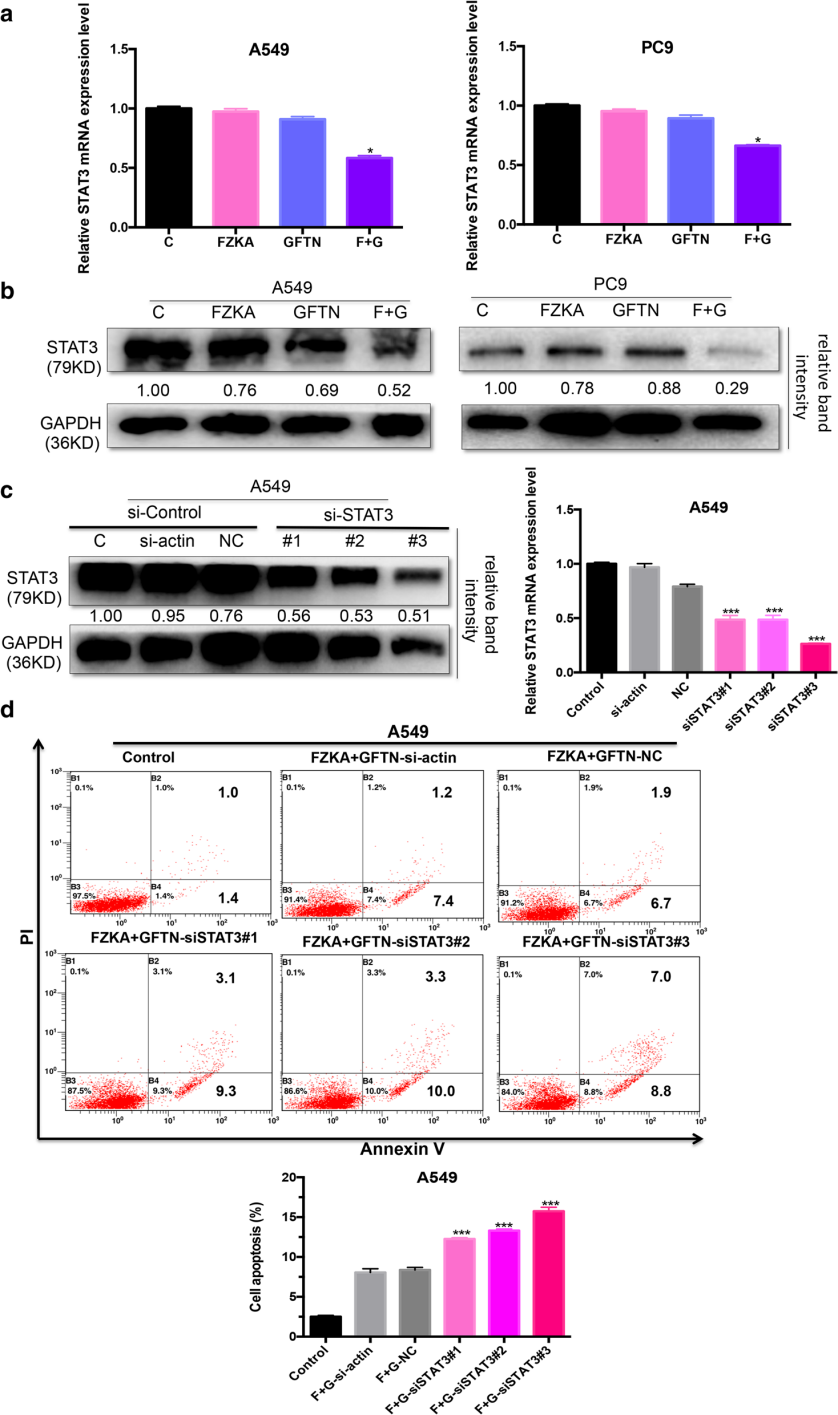
STAT3 mediates the function of FZKA on enhancing Gefitinib-induced cell apoptosis. **a**, **b** The expression of STAT3 was obviously inhibited in the combined group when treated with FZKA and Gefitinib at both mRNA and protein levels. qRT-PCR and western blot were done to test the expression of STAT3 at mRNA and protein levels in lung cancer cells (A549 and PC9) treated with FKZA, Gefitinib, or FZKA combined with Gefitinib for 24 h, respectively. *p < 0.05; one-way ANOVA. The qRT-PCR data were analyzed with 2^−ΔΔCt^ for relative changed in gene expression. GAPDH was used as an internal control. Densitometric analysis was performed using ImageJ. **c** STAT3 was silenced in A549 cells transfected with siSTAT3 (#1, 2 and 3). Western blot was performed to verity the protein expression of STAT3 after transfecting with siSTAT3 (left panel). qRT-PCR was performed to verify the STAT3 expression at mRNA level by transfecting with siSTAT3 (right panel). ***p < 0.0001; Student’s t-test; siSTAT#1, 2 and 3 was compared to NC, respectively. The qRT-PCR data were analyzed with 2^−ΔΔCt^ for relative changed in gene expression. GAPDH was used as an internal control. Densitometric analysis was performed using ImageJ. **d** Cell apoptosis was greatly induced in the siSTAT3 group when treated with the combination of FZKA with Gefitinib. Flow cytometry of cell apoptosis was performed in A549 cells when treated with FZKA combined with Gefitinib as well as transfecting with siGAP, or NC, or siSTAT3. ***p < 0.0001; one-way ANOVA. Data represent mean ± SD of three independent experiments

To further identify the upstream critical molecule which mediates the process of FZKA enhanced Gefitinib-induced lung cancer cell apoptosis. Signal transducer and activator of transcription 3 (STAT3), a transcription factor, plays an important role in cancer cell apoptosis, was found to be inhibited by FZKA, GFTN, and much more in the combination group (FZKA combined with GFTN), both at mRNA and protein levels (Fig. 5a, b). To elucidate whether the additive effect of FZKA and Gefitinib was mediated by STAT3. We silenced the expression of STAT3 by transfecting A549 cells with siSTAT3 (#1,2,3). The siSTAT3 #3 demonstrated greatest efficacy as shown in Fig. 5c. Then we treated cells with FZKA and Gefitinib, and then performed flow cytometry analysis of cell apoptosis. Our results showed the most increase percentage of cell apoptosis in the siSTAT3 #3 group, which has the most inhibition effect of STAT3 expression (Fig. 5d). Therefore, our findings suggested that STAT3 mediates the function of FZKA on enhancing Gefitinib-induced cell apoptosis.

### FZKA enhances the effect of Gefitinib-induced cell apoptosis through Bcl-2 family in vivo

**Fig. 6:**
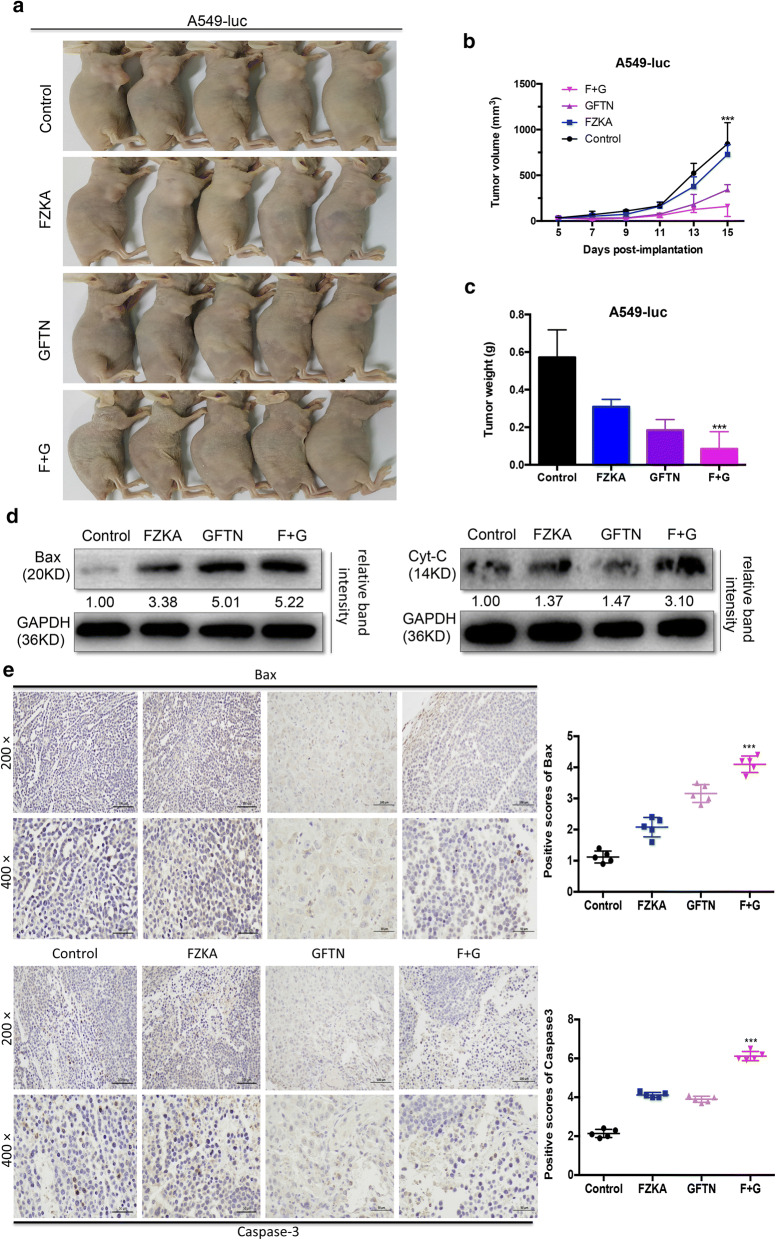
Bcl-2 family mediates the enhanced effect of Gefitinib-induced cell apoptosis by FZKA in vivo. **a**–**c** Tumor growth was the most suppressed in the combined group treated with FZKA and Gefitinib. Animal model was constructed and tumor volume as well as tumor weight was measured as indicated. ***p < 0.0001; one-way ANOVA. **d** Bax and Cyt-*C* was the most induced in the mice tumor tissues treated with FZKA together with Gefitinib. Western blot was performed to detect the protein level of Bax and Cyt-*C* in the mice tumor tissues treated with FZKA, Gefitinb, or FZKA combined with Gefitinib, respectively. GAPDH was used as a loading control. Densitometric analysis was performed using ImageJ. **e** Bax and Caspase-3 was overexpressed in the combined group treated with FZKA and Gefitinib. Immunohistochemistry was carried out to measure the expression of Bax and Caspase-3 in mice tumor tissues treated with FZKA, Gefitinib, or FZKA combined with Gefitinib, respectively. ***p < 0.0001; one-way ANOVA

We showed that the combination of FZKA and Gefitinib had a better inhibition effect on lung cancer tumor growth in vivo, compared to FZKA alone or Gefitinib alone group (Fig. 6a). The tumor volume and weight was much more inhibited in the combination group (Fig. 6b, c). We further extract protein from the tumor tissues and performed western blot to detect the protein expression of Bax (a pro-apoptotic protein of Bcl-2 family) and Cyt-*C* (a key protein of cell apoptosis in the mitochondrial pathway). Our results showed that both Bax and Cyt-*C* were upregulated obviously in the combination group (Fig. 6d). Interestingly, the same result of Bax protein expression by immunohistochemistry was also observed in the mice tumor tissues. Moreover, compared with FZKA alone or Gefitinib alone group, Caspase-3 protein expression was also significantly upregulated in the combination group (Fig. 6e). Taken together, these in vivo findings further emphasized the key role of Bcl-2 family and mitochondrial apoptotic pathway in mediating the synergistic effect of FZKA combined with Gefitinib in lung cancer.

### FZKA sensitizes the effect of Gefitinib-induced cell apoptosis in lung cancer via mitochondrial pathway

**Fig. 7:**
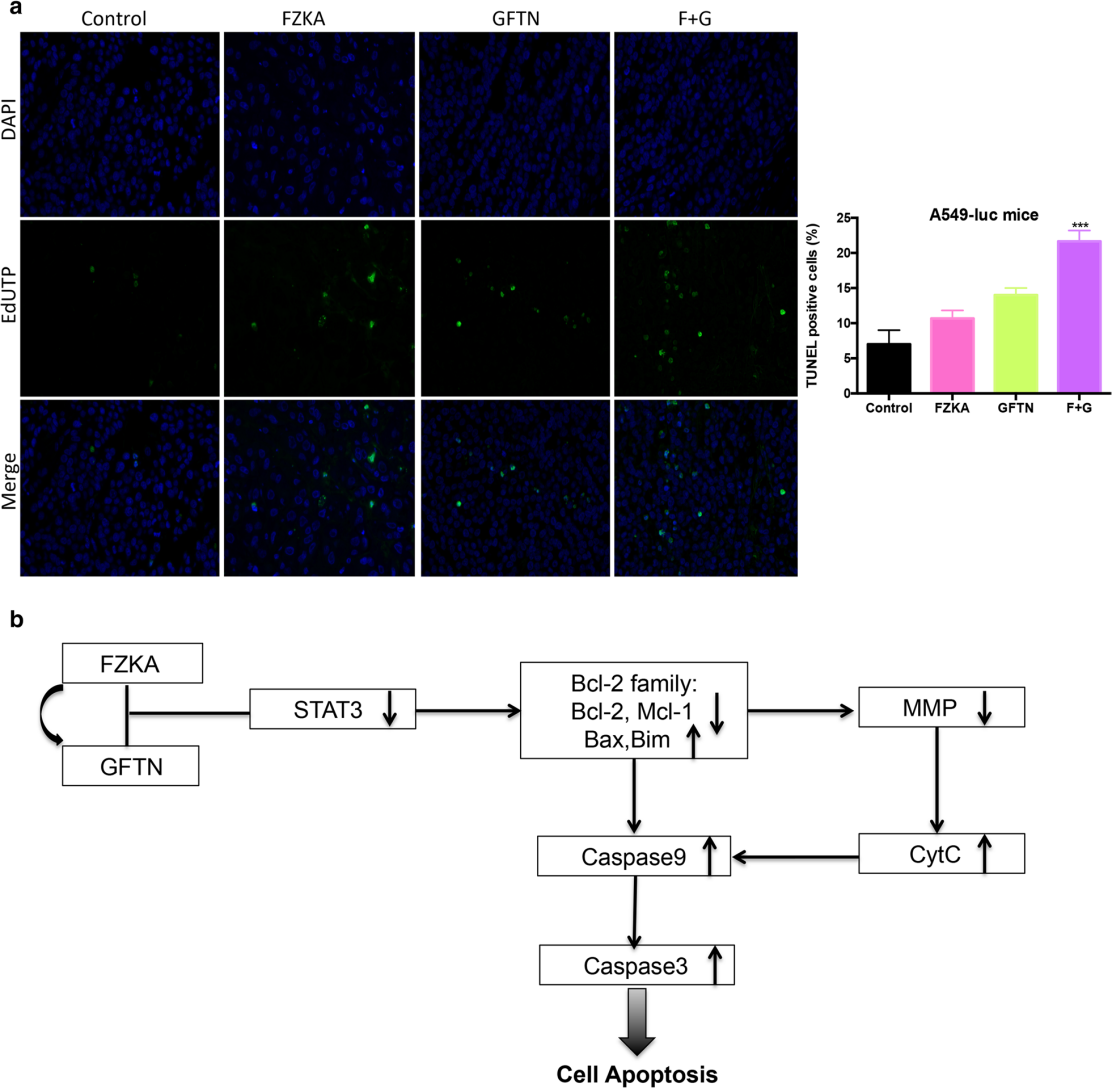
FZKA sensitizes the effect of Gefitinib-induced cell apoptosis in lung cancer via Bcl2/mitochondrial pathway. **a** Cell apoptosis was induced the most in the combined group treated with FZKA and Gefitinib in vivo. TUNEL assay was performed to detect cell apoptosis in mice tumor tissues treated with FZKA, Gefitinib, or FZKA combined with Gefitinib, respectively. The magnification is 400×. ***p < 0.0001; one-way ANOVA. **b** A schematic diagram shows that FZKA has a synergistic effect on Gefitinib-induced cell apoptosis in lung cancer through inactivation of STAT3, followed by reducing anti-apoptotic proteins such as Bcl-2 and Mcl-1, while inducing pro-apoptotic proteins including Bax and Bim. This reduces MMP, leading to the release of Cyc-C from mitochondria to cytoplasm and then activates Caspase-9, resulting in activation of Caspase-3 and cell apoptosis

To reconfirm the effect of FZKA sensitizing Gefitinib-induced cell apoptosis in lung cancer, we did TUNEL assay in the mice tumor tissues. And our results showed that the combination group had the most TUNEL positive cells compared to FZKA alone or Gefitinib alone group (Fig. 7a). Therefore, we drew a mechanism graph showing there is a synergistic effect of FZKA and Gefitinib in promoting lung cancer cell apoptosis. In the process, STAT3 is an upstream key molecular and mediates the effect of cell apoptosis by targeting Bcl-2 family. Then they lead to the decrease of MMP, resulting in the release of Cyt-*C* from mitochondria to cytoplasm. Afterwards, Caspase-9 was activated, and then leading to the activation of Caspase-3, finally resulting in cell apoptosis (Fig. 7b). All in all, FZKA sensitizes the effect of Gefitinib-induced cell apoptosis in lung cancer in a mitochondrial pathway.

## Discussion

In clinic, acquired drug resistance to Gefitinib is a main obstacle for treating NSCLC patients. Our previous clinical study has shown FZKA decoction could prolong the PFS and MST as well as reduce the toxic effect when combined with Gefitinib in treating NSCLC patients. However, the underlying mechanism is about to be elucidated. Here in the present study, we are proposed to uncover the mechanisms by which FZKA decoction affects Gefitinib-induced apoptosis in lung cancer.

In our recent study, we found that FZKA could induce lung cancer cell apoptosis [13]. Therefore, we hypothesized that FZKA might enhances the effect of Gefitinib in lung cancer by regulating cell apoptosis. Actually, there were study reporting that Chinese Herbal Medicine (CHM) Yangfei Kongliu Formula (YKF) combined with chemotherapy cisplatin could inhibit the growth and metastasis of lung cancer through regulating TGF-β1, indicating the important role of CHM in treating lung cancer patients in clinic [16]. Another study reported that Homeopathic Psorinum 6× could trigger apoptosis in lung cancer A549 cells thorough regulating p53, Caspase-3, Bax and Bcl-2 [17]. It is well known that there are two classical pathways of cell apoptosis including mitochondrial and death receptor pathways [18, 19]. The decrease of mitochondrial membrane potential (MMP) and increase of Caspase activity induced by the openness of mitochondrial outer membrane permeability (MOMP) are two main characters of mitochondrial apoptotic pathway [20]. In our study, we found that the induction of cell apoptosis by Gefitinib could be enhanced by FZKA treatment in lung cancer both in vitro and in vivo. Meanwhile, the activity of Caspase-9, a representative molecular in mitochondrial apoptotic pathway, was significantly upregulated and activated by the treatment of FZKA together with Gefitinib, compared to FZKA alone or Gefitinib alone group. This data suggested that mitochondrial pathway plays an important role in FZKA enhanced effect of Gefitinib-induced cell apoptosis in lung cancer.

Bcl-2 family is a large apoptosis regulatory protein that modulates the mitochondrial pathway [21]. Bax, a pro-apoptotic protein from Bcl-2 family, can transfer to mitochondrial outer membrane along with cytoplasm and thus enhance the permeability of mitochondrial membrane, leading to the release of cytochrome *C* (Cyc-*C*) and other pro-apoptotic molecules from inter-membranous space to cytosol, resulting in the activation of downstream Caspases [22, 23]. The increase of Bax could overcome the regulation by Bcl-2 of  mitochondrial  membrane  protein  permeability [24]. In our study, we found that the combination group (FZKA together with Gefitinib) had the most increase expression of Bax and Cyt-*C*, compared to FZKA alone or Gefitinib alone group, both in vitro and in vivo. In addition, Bcl-2 and Mcl-1, two anti-apoptotic proteins from Bcl-2 family, were also altered accordingly. And Bim, another pro-apoptotic protein from Bcl-2 family, was also the most upregulated in the combination group. These data presented herein suggested that Bcl-2 family mediates the FZKA-enhanced effect of Gefitinib-induced lung caner cell apoptosis, and reconfirmed in a mitochondria-dependent manner.

As we all known that transcription factor signal transducer and activator of transcription 3 (STAT3) is closely associated with apoptosis in cancer. It could regulate cell survival by inducing Bcl-2 to repress apoptosis [25]. Therefore, inhibition of STAT3 could induce cancer cell apoptosis [26]. Innovative inhibitors of STAT3 would provide novel strategies for treating human cancers [27]. Intriguingly, our study found that FZKA could inhibit STAT3 expression and the combination of FZKA with Gefitinib had the most inhibition effect on the expression of STAT3. Importantly, when we silenced STAT3 and treated cells with both FZKA and Gefitinib, cell apoptosis were much more induced compared to control group, indicating the critical role of STAT3 in the induction of lung cancer cell apoptosis. Therefore, our findings provides a valid evidence that FZKA might function as a STAT3 inhibitor when treat lung cancer patients. And since Bcl-2 is one of the targets of STAT3, the inhibition of STAT3 will lead to the suppression of Bcl-2, and thus resulting in cell apoptosis promotion. In total, our results reconfirmed that FZKA enhances Gefitinib-induced cell apoptosis via mitochondrial pathway. Our findings uncover a novel mechanism by which FZKA treat lung cancer patients and how FZKA sensitizes or enhances the effect of Gefitinib, which provides promising strategy to resolve the issue of acquired drug resistance to Gefitinib in treating lung cancer patients in clinic.

In our study, we investigated the combination effects of FZKA and Gefitinib on the cell apoptosis in human lung cancer both in vitro and in vivo. Our results show that FZKA enhances the effect of Gefitinib-induced lung cancer cell apoptosis via inhibition of STAT3, followed by increasing protein expression of Bax and Bim, concomitantly decreasing Bcl-2 and Mcl-1 protein levels, leading to decrease of MMP and release of Cyt-*C* from mitochondria to cytoplasm, and significantly activating Caspase-9 and Caspase-3. Totally, FZKA enhances the effect of Gefitinib-induced lung cancer cells in a mitochondrial pathway, which provides a clear mechanism by which FZKA functions in treating lung cancer patients. Our study indicates novel evidences about the clinical use of FZKA together with Gefitinib in lung cancer patients.

## Conclusions

This study shows that FZKA decoction enhances the effect of Gefitinib-induced lung cancer cells in a mitochondrial pathway through STAT3 and Bcl-2 family (Fig. 7b). Both our in vitro and in vivo experiments provides a novel mechanism by which the FZKA decoction enhances the cell apoptosis induction of gefitinib, indicating an alternative therapeutic strategy for the treatment of lung cancer patients with drug resistance to gefitinib.

## Supplementary information

**Additional file 1: Table S1.** Sequences of all primers (qRT-PCR) and siRNAs. **Figure S1.** HPLC chromatograms in different drinks of FZKA decoction have similar patterns. **Figure S2.** STAT3 was inhibited both at protein and mRNA levels by treating with siSTAT3 in PC9 cells. **Figure S3.** Flow cytometry analysis of cell apoptosis data showing the gating strategy.

## Data Availability

The datasets used and/or analyzed during the current study are available from the corresponding author on reasonable request.

## Abbreviations

CHM: Chinese herbal medicine
FZKA: Fuzheng Kang-Ai
GFTN: Gefitinib
TUNEL: TdT-mediated dUTP Nick-End Labeling
MMP: Mitochondrial membrane potential
IHC: Immunohistochemistry
EGFR-TKI: Epidermal growth factor receptor tyrosine kinase
NSCLC: Non-small cell lung cancer
PFS: Progression-free survival
DCR: Disease control rate
MST: Median survival time
HPLC: High performance liquid chromatography
Mcl-1: Myeloid cell leukemia-1
Cyt-*C*: Cytochrome *C*
BLI: Bioluminescence imaging
PVDF: Transferred onto polyvinylidene fluoride
qRT-PCR: Quantitative real-time polymerase chain reaction
YKF: Yangfei Kongliu Formula
MOMP: Mitochondrial outer membrane permeability
STAT3: Signal transducer and activator of transcription 3
