# Supplementary material for: Fuzheng Kang-Ai decoction enhances the effect of Gefitinib-induced cell apoptosis in lung cancer through mitochondrial pathway
Source: Cancer Cell Int. 2020 May 24;20:185. doi: 10.1186/s12935-020-01270-3 (PMC7247206; doi:10.1186/s12935-020-01270-3)
Supplement: Supplementary file 1 — Additional file 1: Table S1. Sequences of all primers (qRT-PCR) and siRNAs. Figure S1. HPLC chromatograms in different drinks of FZKA decoction have similar patterns. Figure S2. STAT3 was inhibited both at protein and mRNA levels by treating with siSTAT3 in PC9 cells. Figure S3. Flow cytometry analysis of cell apoptosis data showing the gating strategy. [file 12935_2020_1270_MOESM1_ESM.docx]

**Additional Tables**

Table S1. Sequences of all primers (qRT-PCR) and siRNAs

mRNA Primer sequence

Bcl-2 Forward: AGAAGTCTGGGAATCGATCTGG

Reverse: AACTTCCCAATGAATCAGGAGTC

Mcl-1 Forward: AAGCAAGTGGCAAGAGGATTATG

Reverse: TCCACCCTACCATCTTCACTAAAT

Bax Forward: CCCGAGAGGTCTTTTTCCG

Reverse: TGAGCACCAGTTTGCTGGC

Bim Forward: AGAGTTGCGGCGTATTGGA

Reverse: GGTCTTCGGCTGCTTGGTAA

STAT3 Forward: GCGTCCAGTTCACTACTAAAGTCAG

Reverse: CCAGAGTCTTTGTCAATGCACAC

GAPDH Forward: GAACGGGAAGCTCACTGG

Reverse: GCCTGCTTCACCACCTTCT

siSTAT3#1 Target sequence: CATCTGCCTAGATCGGCTA

siSTAT3#2 Target sequence: AGTCAGGTTGCTGGTCAAA

siSTAT3#3 Target sequence: CCGTGGAACCATACACAAA

**Additional Figures**

**Figure S1. HPLC chromatograms in different drinks of FZKA decoction have similar patterns.** The water extraction of the compound prescriptions of the different groups of the FZKA decoction was qualitatively analyzed by HPLC method as described in Section 2. Conditions: column: C18 column (250 × 4.6 mm, 5 𝜇m); rate: 1.0 mL/min; column temperature: 30∘ C; injection volume: 10 𝜇L. Four batches of FZKA decoction water extracts including a mixture of three different batches (FZKA 1–4) are presented.


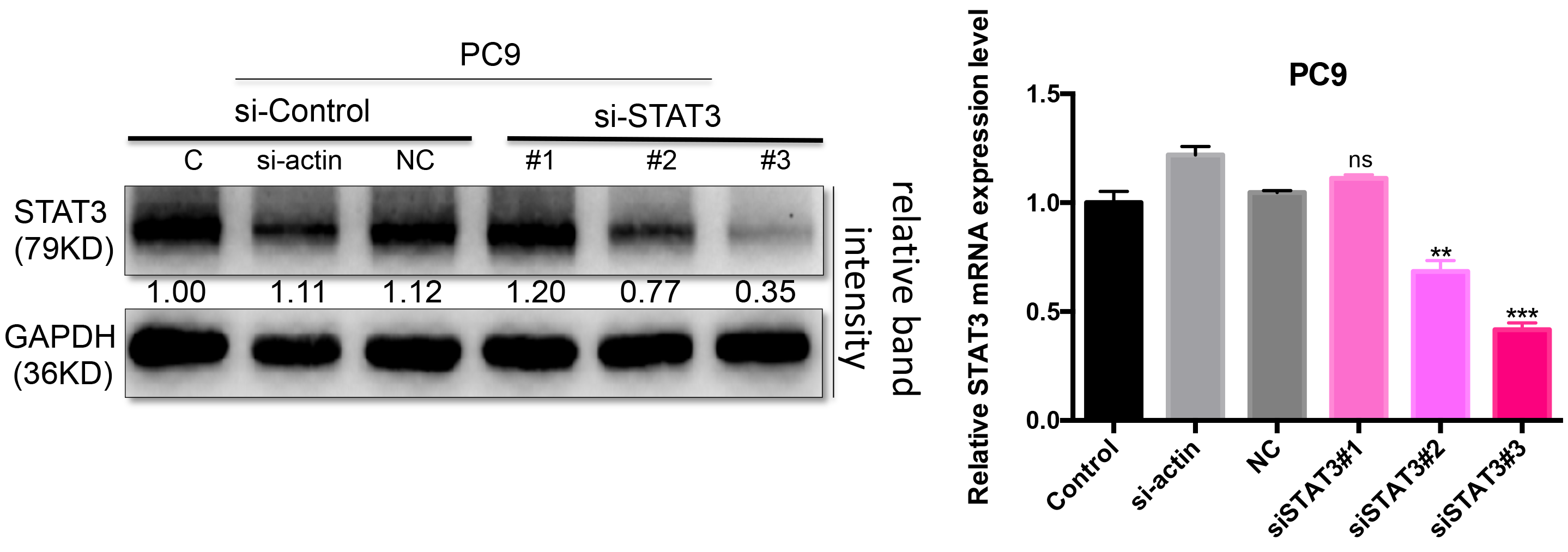


**Figure S2. STAT3 was inhibited both at protein and mRNA levels by treating with siSTAT3 in PC9 cells.** STAT3 was silenced in PC9 cells transfected with siSTAT3 (#1, 2 and 3). Western blot was performed to verity the protein expression of STAT3 after transfecting with siSTAT3 (left panel). qRT-PCR was performed to verify the STAT3 expression at mRNA level by transfecting with siSTAT3 (right panel). ns, not significant. **, *p*<0.01, ***, *p*<0.001; Student’s t-test; siSTAT#1, 2 and 3 was compared to NC, respectively. The qRT-PCR data were analyzed with 2^−ΔΔCt^ for relative changed in gene expression. GAPDH was used as an internal control. Densitometric analysis was performed using ImageJ.

**Figure S3. Flow cytometry analysis of cell apoptosis data showing the gating strategy.**
